# Novel patient-derived models of desmoplastic small round cell tumor confirm a targetable dependency on ERBB signaling

**DOI:** 10.1242/dmm.047621

**Published:** 2022-01-27

**Authors:** Roger S. Smith, Igor Odintsov, Zebing Liu, Allan Jo-Weng Lui, Takuo Hayashi, Morana Vojnic, Yoshiyuki Suehara, Lukas Delasos, Marissa S. Mattar, Julija Hmeljak, Hillary A. Ramirez, Melissa Shaw, Gabrielle Bui, Alifiani B. Hartono, Eric Gladstone, Siddharth Kunte, Heather Magnan, Inna Khodos, Elisa De Stanchina, Michael P. La Quaglia, Jinjuan Yao, Marick Laé, Sean B. Lee, Lee Spraggon, Christine A. Pratilas, Marc Ladanyi, Romel Somwar

**Affiliations:** 1 Department of Pathology, Memorial Sloan Kettering Cancer Center, New York, NY 10065, USA; 2 Human Oncology and Pathogenesis Program, Memorial Sloan Kettering Cancer Center, New York, NY 10065, USA; 3 Anti-tumor Assessment Core Facility, Molecular Pharmacology Program, Memorial Sloan Kettering Cancer Center, New York, NY 10065, USA; 4 Gerstner School of Graduate Studies, Memorial Sloan Kettering Cancer Center, New York, NY 10065, USA; 5 Tulane University School of Medicine, New Orleans, LA 70118, USA; 6 Department of Pediatrics, Memorial Sloan Kettering Cancer Center, New York, NY 10065, USA; 7 Department of Surgery, Memorial Sloan Kettering Cancer Center, New York, NY 10065, USA; 8 Division of Pediatric Oncology, Sidney Kimmel Comprehensive Cancer Center at Johns Hopkins, Baltimore, MD 21287, USA

**Keywords:** EWSR1-WT1, DSRCT PDX, EGFR, Sarcoma proteomics

## Abstract

Desmoplastic small round cell tumor (DSRCT) is characterized by the t(11;22)(p13;q12) translocation, which fuses the transcriptional regulatory domain of EWSR1 with the DNA-binding domain of WT1, resulting in the oncogenic EWSR1-WT1 fusion protein. The paucity of DSRCT disease models has hampered preclinical therapeutic studies on this aggressive cancer. Here, we developed preclinical disease models and mined DSRCT expression profiles to identify genetic vulnerabilities that could be leveraged for new therapies. We describe four DSRCT cell lines and one patient-derived xenograft model. Transcriptomic, proteomic and biochemical profiling showed evidence of activation of the ERBB pathway. Ectopic expression of EWSR1-WT1 resulted in upregulation of ERRB family ligands. Treatment of DSRCT cell lines with ERBB ligands resulted in activation of EGFR, ERBB2, ERK1/2 and AKT, and stimulation of cell growth. Antagonizing EGFR function with shRNAs, small-molecule inhibitors (afatinib, neratinib) or an anti-EGFR antibody (cetuximab) inhibited proliferation of DSRCT cells. Finally, treatment of mice bearing DSRCT xenografts with a combination of cetuximab and afatinib significantly reduced tumor growth. These data provide a rationale for evaluating EGFR antagonists in patients with DSRCT.

This article has an associated First Person interview with the joint first authors of the paper.

## Abstract (precis)

**Summary:** Novel models of desmoplastic small round cell tumor (DSRCT) reveal a role for the ERBB pathway in regulating growth of this sarcoma and provide a rationale for evaluating EGFR antagonists in patients with DSRCT.

## INTRODUCTION

Desmoplastic small round cell tumor (DSRCT) is a devastating sarcoma of primitive histology that affects pediatric and adolescent patients (Gerald et al., 1991). Tumors are characterized by the recurrent chromosomal translocation t(11;22)(p13;q12) (Sawyer et al., 1992; Violet Shen et al., 1992), which results in the fusion of the first 7, 9, or 10 exons of *EWSR1* to exon 8 of *WT1*, coupling the transcriptional regulatory domain of *EWSR1* and the DNA-binding domain of *WT1* (Gerald et al., 1995; Gerald and Haber, 2005; Ladanyi and Gerald, 1994). Patients with DSRCT have poor overall outcomes despite initial responses to aggressive treatments, including surgery, chemotherapy and radiation (Hayes-Jordan et al., 2016). Complete surgical resection is challenging due to the abundance of disseminated lesions at multiple peritoneal sites at the time of diagnosis (Hayes-Jordan et al., 2016). In a report on the longitudinal experience over 30 years with a cohort of 66 patients, overall survival was a dismal 15% at 5 years (Lal et al., 2005).

The development of targeted therapy for DSRCT and other sarcomas driven by chimeric transcription regulators has lagged behind that for other solid tumors. One of the main reasons is that targeting the primary drivers with small molecules that need to penetrate the nuclear compartment is challenging due to the non-enzymatic nature of chimeric transcription regulators and the complexity of their binding to DNA. To improve clinical outcomes for this aggressive adolescent and young adult sarcoma, it will be necessary to identify alternative therapeutic strategies.

Protein kinases may represent genetic vulnerabilities that can be exploited as potential therapeutic targets for DSRCT. As kinase signaling is central to cell proliferation and survival, it is likely that these aberrant transcription factors achieve their effects on cell growth through the dysregulation of specific kinase signaling pathways. Identifying these upregulated kinases, which remain poorly defined for DSRCT and other sarcomas, may highlight therapeutic vulnerabilities that are more readily targetable than the fusion protein themselves. We have previously demonstrated the value of this approach in another translocation-positive sarcoma, alveolar soft-part sarcoma (ASPS), where the ASPL-TFE3 fusion upregulates the MET receptor tyrosine kinase (RTK), creating a targetable dependency on MET signaling (Kobos et al., 2013; Tsuda et al., 2007). In addition, we have previously demonstrated the sensitivity of a subset of synovial sarcoma (SS) to inhibition of PDGFRA, a kinase often upregulated in this disease (Ho et al., 2012). In Ewing sarcoma (ES) and SS, some patients have been observed to respond to IGF1R inhibitors and pazopanib (a multi-targeted RTK inhibitor), respectively (Pappo et al., 2011; Olmos et al., 2010; Sleijfer et al., 2009; Casanova et al., 2017), indicating targetable dependencies on kinase signaling in these two sarcomas, although the precise mechanisms of those dependencies have remained elusive.

We hypothesize that, similar to SS, ES and ASPS, DSRCT has unique dependencies on kinases that can be exploited for therapy. Although DSRCT and the oncogenic transcription factor were identified ∼30 years ago, studying the biology of this tumor to identify potential therapeutic targets has been limited by the paucity of preclinical disease models. Indeed, there is only one published, widely available DSRCT cell line, JN-DSRCT-1, first described in 2002 (Nishio et al., 2002). The development of effective targeted therapy requires disease models such as isogenic and patient-derived cell lines and xenograft models that maintain their original characteristics and are easily manipulated for molecular genetic approaches and drug development studies. Our goal in this study was to generate new patient-derived DSRCT cell lines and xenograft models and to use these to identify kinases that may serve as therapeutic targets. We report here four new fully characterized cell lines and one patient-derived xenograft (PDX) model and show that the ERBB pathway is essential for growth of DSRCT cells and can be exploited for therapy. Importantly, we report the first orthotopic xenograft tumor models of DSRCT and demonstrate significant reduction in tumor burden with EGFR-targeted therapies.

## RESULTS

### Transcriptome profiling identifies the ERBB pathway as activated in DSRCT tumor samples

**Fig. 1.**
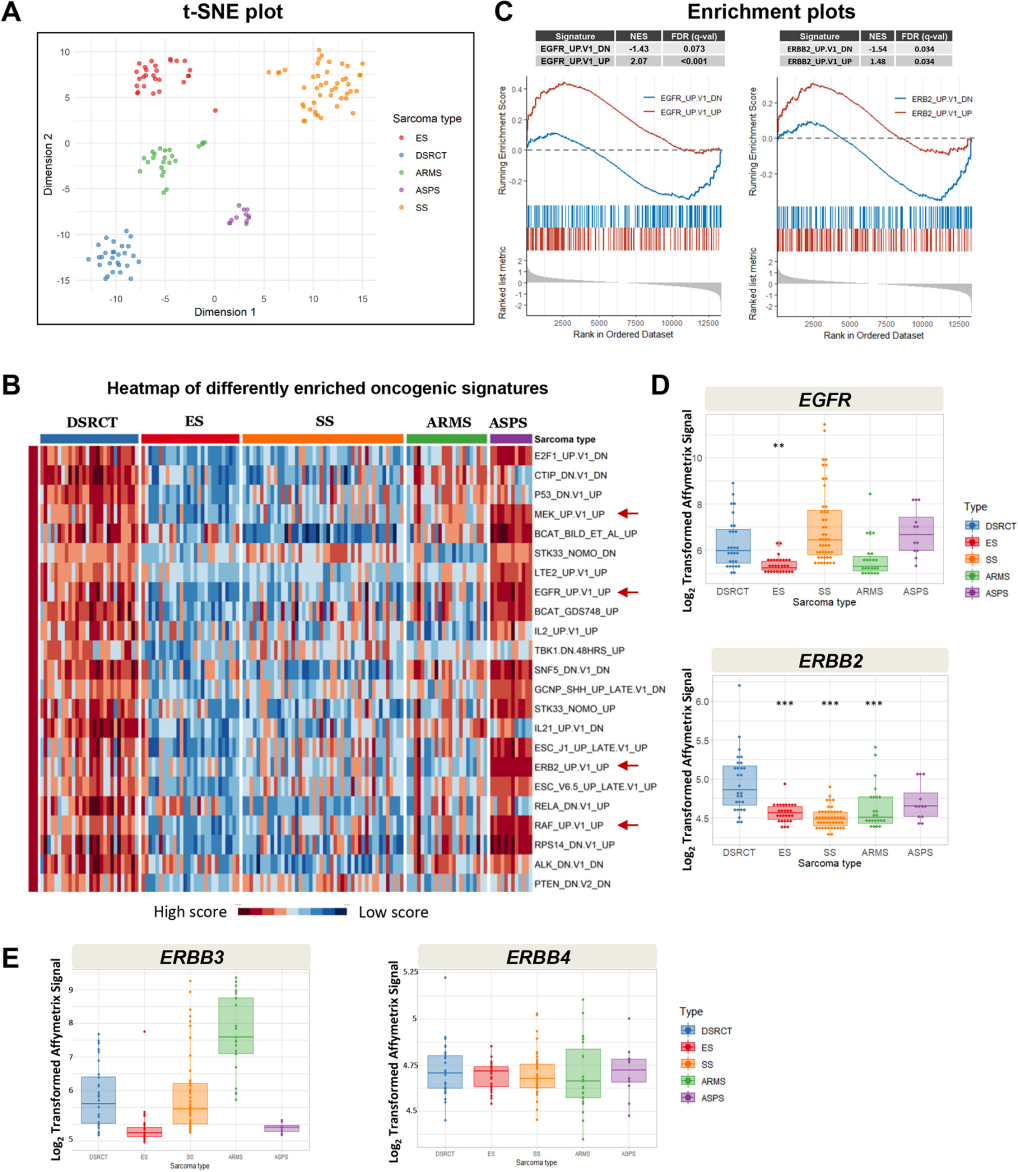
**Analysis of mRNA expression data in 137 sarcoma samples reveals activation of the ERBB pathway in DSRCT.** (A) t-SNE analysis. Sarcoma type-specific clustering of samples supports intergroup differential expression analysis. (B) GSVA heatmap. GSVA is a non-parametric unsupervised method that allows the assessment of gene set enrichment in each individual sample. ‘Oncogenic signatures’ from the Molecular Signature Database were queried. Enrichment scores were then compared in sarcoma types using linear regression, and the top gene sets differentially enriched in DSRCT relative to other sarcomas are presented as a heatmap. (C) GSEA analysis was also performed comparing DSRCT to ES samples using oncogenic signatures. Enrichment plots for ERBB-related pathways are presented with NESs and FDR (*q*-value). (D) Box plots of the expression of *EGFR* (top) and *ERBB2* (bottom). (E) Box plots of the expression of ERBB3 and ERBB4. Box plots show the median. Log_2_-transformed raw values are presented. ***adjusted *P*<0.0001, **adjusted *P*=0.008 (unpaired, one-tailed Student's *t*-test). **, *** refer only to pairwise comparisons where expression is higher in DSRCT. ARMS, alveolar rhabdomyosarcoma; ASPS, alveolar soft-part sarcoma; DSRCT, desmoplastic small round cell tumor; ES, Ewing sarcoma; FDR, false discovery rate; GSEA, gene set enrichment analysis; GSVA, Gene Set Variability Analysis; NES, normalized enrichment score; SS, synovial sarcoma; t-SNE, t-distributed stochastic neighbor embedding.

To discover oncogenic pathways that may be selectively activated in DSRCT, we mined legacy microarray-based mRNA expression profiling data of 137 translocation-positive sarcoma samples (Filion et al., 2009). This dataset comprised 28 DSRCTs, 28 ESs, 23 fusion-positive alveolar rhabdomyosarcomas (ARMSs), 46 SSs and 12 alveolar soft-part sarcomas (ASPSs), all fusion verified by clinical RT-PCR assays. These sarcomas are characterized by distinct chromosomal translocations that produce chimeric transcription factors with unique composition, resulting in specific signatures of pathway activation. An unbiased clustering of the expression data using t-distributed stochastic neighbor embedding (t-SNE) analysis (Van Der Maateen and Hinton, 2008) organized samples into clearly defined groups according to sarcoma type (Fig. 1A), confirming the highly distinctive transcriptomes generated by these aberrant transcription factors.

To determine whether DSRCT has a distinct gene expression profile reflecting activated growth-promoting pathways that could be exploited for therapy, we interrogated the transcriptomes of the samples from the five sarcoma types. We utilized the Gene Set Variability Analysis (GSVA) with oncogenic signature gene sets from Broad MsigDB to identify activated oncogenic pathways. The top oncogenic signatures that were significantly up- or downregulated in DSRCT compared to each of the other sarcomas were ranked by adjusted *P*-value and depicted in Fig. 1B. The finding of EGFR_UP.V1_UP and ERBB2_UP.V1_UP gene set enrichment in DSRCT compared to other sarcomas suggested a role for *EGFR* and *ERBB2* pathway activation in these tumors. In addition, there was significant upregulation of *RAF* (also known as *RAF1*) and *MEK* signatures, two principal ERBB family effector pathways. We next performed gene set enrichment analysis (GSEA) of DSRCT compared to ES samples using oncogenic signatures from the Broad MsigDB database (Mootha et al., 2003; Subramanian et al., 2005). We limited the comparison of DSRCT to ES tumor samples as the latter sarcoma is also driven by an *EWSR1* translocation. This additional analysis showed that the *EGFR* pathway is more active in DSRCT tumors than in ES and underscore the importance of ERBB pathways in DSRCT (Fig. 1C).

Given that EGFR functions either as a homodimer or as a heterodimer with other members of the ERBB family of receptors (Roskoski, 2004), we compared expression of the four ERBB family genes in DSRCT relative to the other four sarcomas in the dataset. Among the five sarcomas, only DSRCT showed relatively high mRNA expression of both *EGFR* (Fig. 1D, top) and *ERBB2* (Fig. 1D, bottom), supporting a potential role for the ERBB family receptors in DSRCT growth. DSRCT tumors did not have a significantly higher level of expression of *ERBB3* or *ERBB4* mRNA compared to the other sarcomas (Fig. 1E).

### Generation of novel tumorigenic DSRCT cell lines

**Fig. 2.**
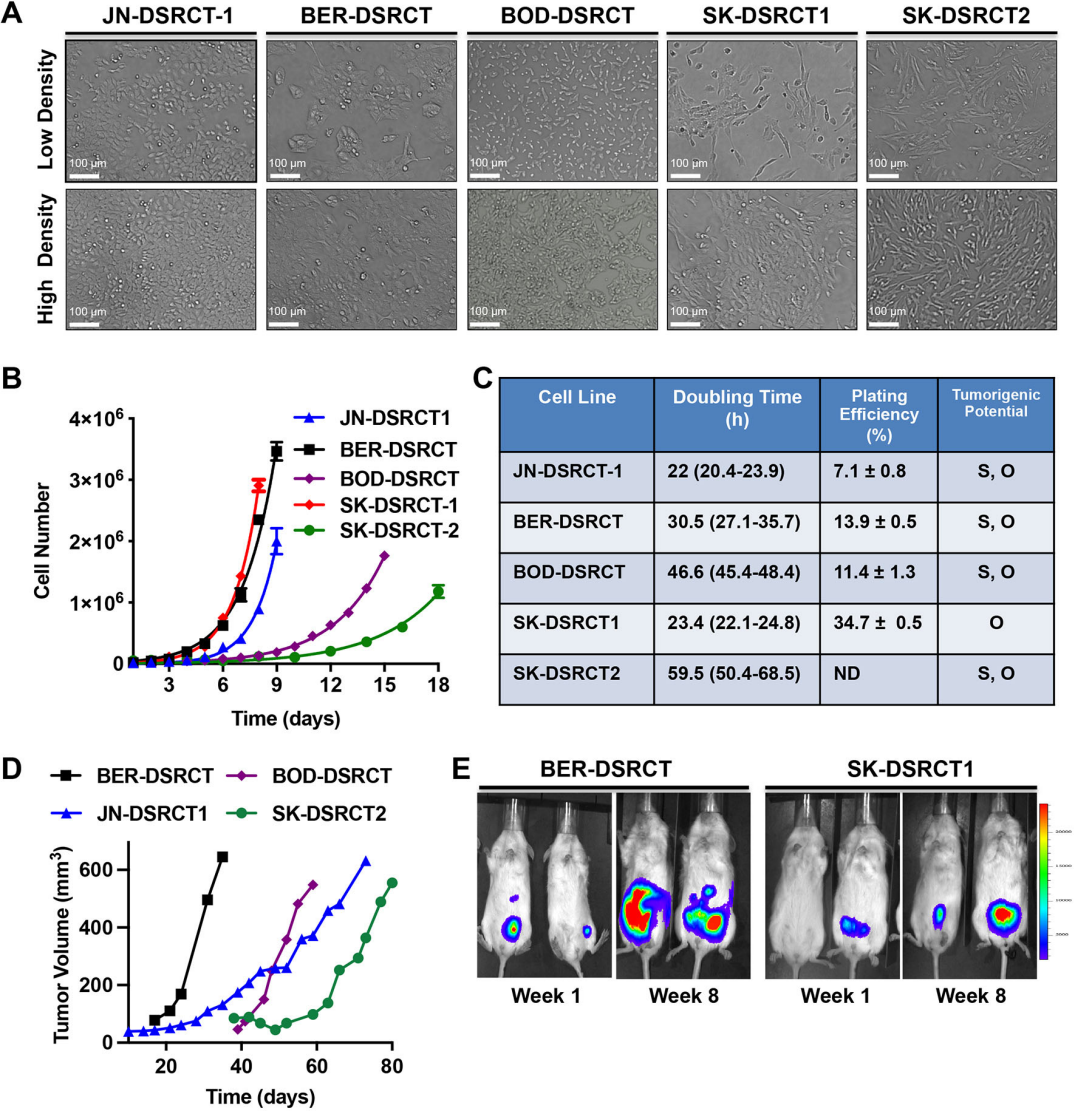
**Generation and characterization of novel DSRCT preclinical models.** (A) Phase-contrast images of DSRCT cell lines (100× magnification). (B) Growth characteristics of DSRCT cell lines in culture. (C) Doubling time, plating efficiency and tumorigenic potential of cells. (D) DSRCT cell lines were implanted into the subcutaneous flank of immunocompromised mice and tumors were measured twice weekly. Data represent the average volume of two tumors per cell line. (E) Cell lines stably expressing a luciferase construct were implanted into the peritoneal cavity of immunocompromised mice, and bioluminescence images were acquired weekly. The first (1 week after implantation) and last (8 weeks after implantation) images are shown. O, orthotopic; S, subcutaneous.

Investigation into the biology of DSRCT has been hindered by a paucity of preclinical disease models. Only the JN-DSRCT-1 cell line is well characterized and widely used (Nishio et al., 2002). To address this obstacle, we sought to develop new patient-derived DSRCT cell lines and xenograft models. We generated two DSRCT cell lines from Memorial Sloan Kettering (MSK) patient samples (SK-DSRCT1, SK-DSRCT2) and characterized two DSRCT cell lines that were previously described but not genomically or biochemically characterized (BER-DSRCT, BOD-DSRCT) (Markides et al., 2013). The morphology of the cells is shown in Fig. 2A, with images obtained at two different degrees of confluence. Patient demographic, clinical information and other details are provided in Table S1. The BOD-DSRCT and BER-DSRCT cells appear as small round cells at low and high densities, similar to JN-DSRCT-1 cells. SK-DSRCT1 and SK-DSRCT2 cells appear as mixed morphology with both cell lines having mixed round and spindle-shaped cells.

We next characterized the growth rate, plating efficiency and tumorigenic potential of the five DSRCT cell lines. The growth rate of cell lines was variable, with JN-DSRCT-1 and SK-DSRCT1 having the shortest doubling time (22 h and 23.4 h, respectively), followed by BER-DSRCT (Fig. 2B,C). The SK-DSRCT2 and BOD-DSRCT cell lines were slowest growing, with doubling times of 59.5 h and 46.6 h, respectively (Fig. 2B,C). In clonogenic assays, the JN-DSRCT-1 cell lines had the lowest plating efficiency (7.1±0.8%) and the SK-DSRCT1 cell line had the highest plating efficiency (34.7±0.5%) (Fig. 2C).

To explore the ability of the cell lines to form xenograft tumors, cells were implanted either in the subcutaneous flank (unlabeled cells) or the peritoneal cavity (cell lines stably expressing a GFP-luciferase cDNA) of immunocompromised mice. Following subcutaneous implantation, all cell lines except SK-DSRCT1 grew into xenograft tumors by day 80 (when the study was terminated) (Fig. 2D). For the orthotopic xenograft models, luciferase-expressing BER-DSRCT and SK-DSRCT1 cells were injected into the peritoneum and then bioluminescence images were acquired weekly. Images taken 1 and 8 weeks after implantation of cells in two mice per cell line are shown in Fig. 2E. The luciferase signals increased over this time, indicating that both BER-DSRCT and SK-DSRCT1 (which did not form subcutaneous tumors) are capable of forming orthotopic xenograft tumors in the peritoneal cavity, which is the most common site of presentation of DSRCT in patients.

### Cytogenetic and genomic characterization of novel DSRCT cell lines

**Fig. 3.**
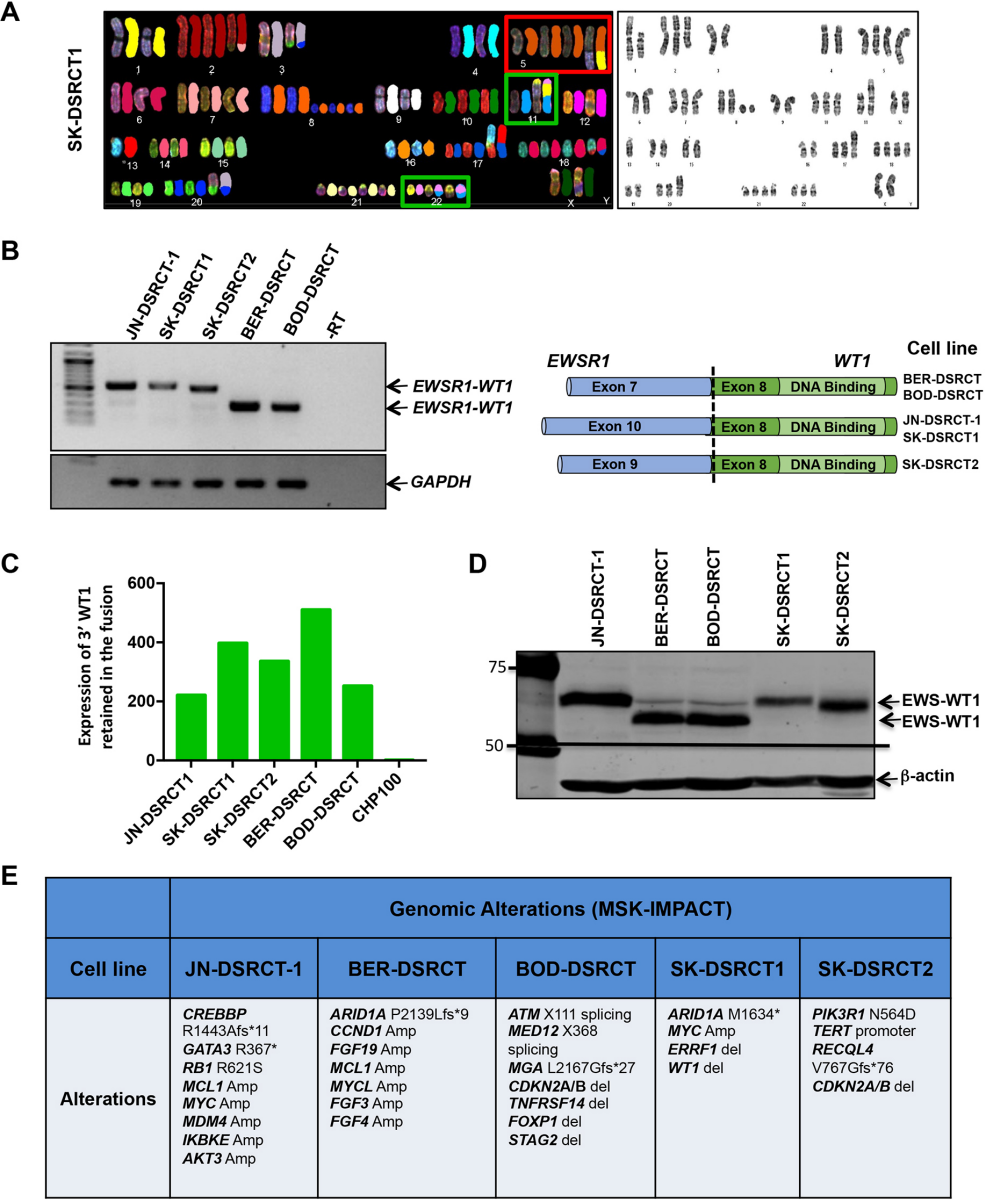
**Cytogenetic, genetic and genomic characteristics of novel DSRCT cell lines.** (A) Spectral (left) or DAPI-banded (right) karyotypes of SK-DSRCT1 cells illustrating the translocation between chromosomes 11 and 22, resulting in the oncogenic chimeric transcription factor. *EWSR1-WT1* is shown in the green boxes; Chr5 polysomy is shown in the red box. The karyotypes of all cell lines are shown in Fig. S1. (B) RT-PCR conducted on five DSRCT cell lines showing expression of the *EWSR1-WT1* fusion (left). PCR amplicons were TOPO cloned and then validated by orthogonal DNA sequencing. The exons of *EWSR1* and *WT1* that were fused in each cell line are shown in the schematic (right). (C) Expression of 3′ *WT1* mRNA retained in the fusion. *EWSR1*-*WT1* mRNA level is expressed relative to that of native *WT1* in CHP100 (ES cell line). (D) Western blot analysis of EWS-WT1 using an anti-WT1 antibody targeting the C-terminal region of WT1 in DSRCT cell lines. (E) DNA was profiled by the MSK-IMPACT platform to identify genomic alterations in cell lines.

Cytogenetic analysis was performed on all cell lines to examine the karyotype. The chromosome spread obtained by either SKY karyotyping or 4′,6-diamidino-2-phenylindole (DAPI) banding are shown in Fig. 3A (SK-DSRCT1 cell line) and in Fig. S1 (all cell lines). More details of the cytogenetic analysis of each cell line are presented in Table S2. The t(11;22)(p13;q12) translocation was identified in all five cell lines, with the typical balanced translocation in three cell lines (JN-DSRCT, BER-DSRCT, BOD-DSRCT). The SK-DSRCT1 and SK-DSRCT2 cell lines had unbalanced t(11;22)(p13;q12). Four of the five DSRCT cell lines had gained either an extra chromosome 5, or had duplication of some region or some parts of it (only BOD-DSRCT showed no copy number alteration of chromosome 5). There were also gains and losses and additional rearrangements that were unique to each cell line (Table S2).

To confirm expression of the chimeric *EWSR1-WT1* gene resulting from the t(11;22)(p13;q12) translocation, we performed RT-PCR using primers for *EWSR1* exon 7 and *WT1* exon 8. Three cell lines had amplicons of similar sizes (JN-DSRCT-1, SK-DSRCT1, SK-DSRCT2), and the remaining two cell lines had a smaller-sized product (Fig. 3B, left). PCR amplicons were cloned into a TOPO vector, expanded in bacteria, and then plasmid DNA from ten colonies was isolated, and the inserts were sequenced to identify the precise exons of *EWSR1* and *WT1* that are fused in each cell line. A schematic of the results is shown in Fig. 3B (right). The cell lines harbored heterogeneous in-frame fusions between *EWSR1* (various exons) and exon 8 of *WT1* (Fig. 3B, right), as has been shown for DSRCT tumors (Liu et al., 2000; La Starza et al., 2013). The BER-DSRCT and BOD-DSRCT cell lines had a fusion between *EWSR1* exon 7 and *WT1* exon 8, which, as we have shown previously, is present in the majority of DSRCT tumors (Gerald et al., 1995). The other cell lines, including JN-DSRCT-1, had *EWSR1-WT1* fusions that occur less frequently in patient samples. Although a ‘9-8’ fusion transcript was predominant in the SK-DSRCT2 cell line, it also expressed a very low level of the ‘7-8’ transcript, presumably from the same allele (Fig. 3B).

We further validated *WT1* expression by quantitative PCR (qPCR) targeting the 3′ part of *WT1* that is retained in the fusion and western blotting using an anti-WT1 antibody that was raised against a peptide mapping at the C-terminal region. The ES cell line CHP100 (*EWSR1-FLI1* fusion) was used as a negative control. As expected, *WT1* mRNA was detected in all DSRCT cell lines and amounted to 200- to 500-fold higher than in CHP100 (Fig. 3C). Western blotting with the C-terminal WT1 antisera confirmed the presence of WT1 in all of the new DSRCT cell lines (Fig. 3D). The band detected is most likely EWSR1-WT1 (hereafter referred to as EWS-WT1) as transcriptomic analysis has shown that the JN-DSRCT-1 cell line does not express wild-type WT1 (Hingorani et al., 2020).

To broadly characterize our novel cell line models, we profiled DNA by next-generation sequencing (NGS) using our MSK-IMPACT (integrated mutation profiling of actionable cancer targets) platform, which captures genomic alterations across 468 genes of proven or potential therapeutic and/or prognostic significance (Cheng et al., 2015). Known COSMIC somatic mutations or truncating mutations in tumor suppressor genes as well as copy number variants (CNVs) are listed for each DSRCT cell line in Fig. 3E. Among the notable findings was a *STAG2* deletion in one sample, a gene known to be recurrently inactivated in an aggressive subset of ESs (Tirode et al., 2014), another *EWSR1*-rearranged sarcoma (El Beaino et al., 2020).

### Proteomic profiling of DSRCT models identifies elevated ERBB family RTK activity

**Fig. 4.**
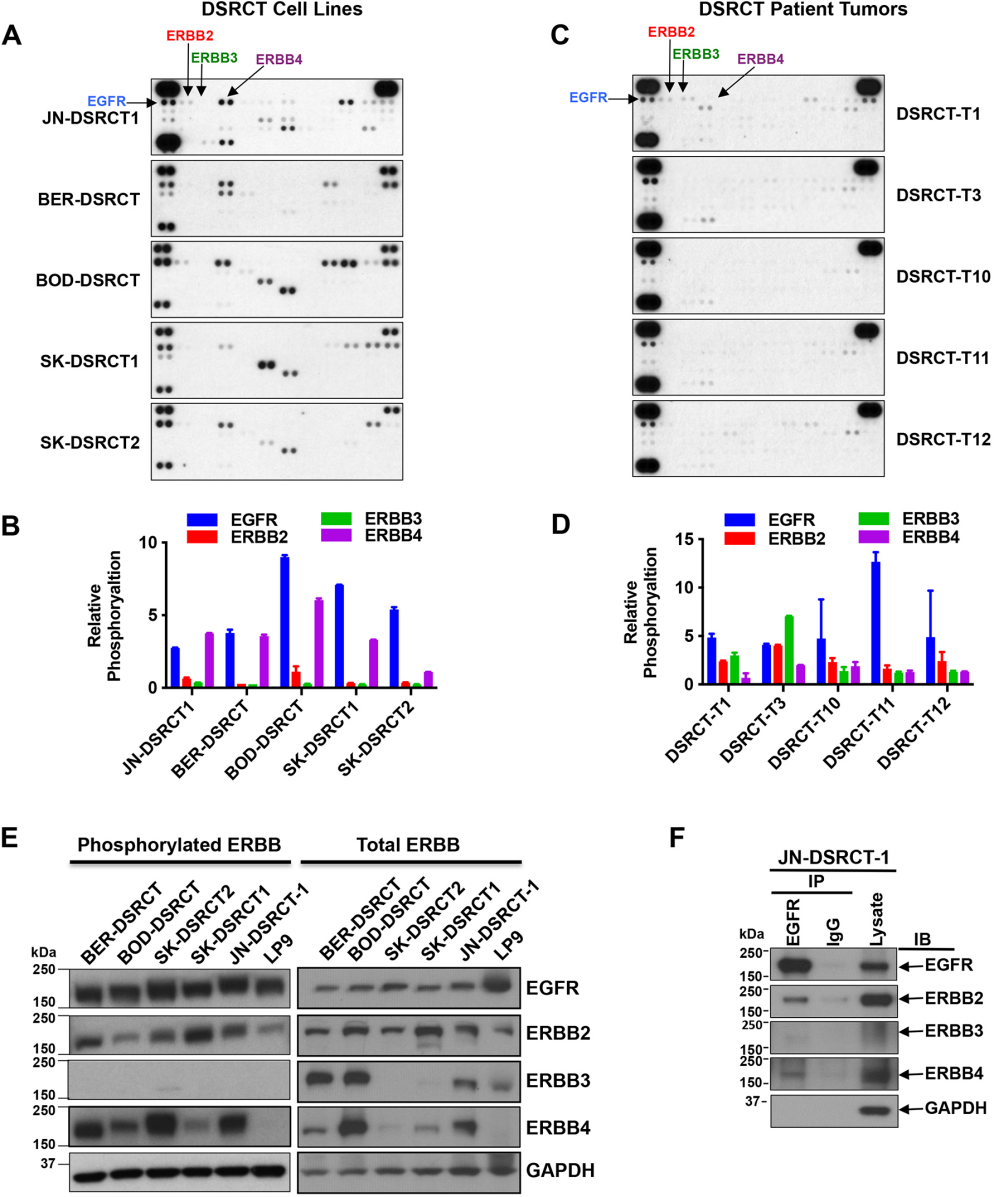
**Expression and activation of ERBB family members in novel DSRCT cell lines.** (A-D) Receptor tyrosine kinase (RTK) arrays were used to profile phosphorylated RTKs in DSRCT cell lines (A,B) or tumors (C,D). Proteomic arrays were imaged on X-ray films and quantitated by densitometry. Values are expressed relative to values obtained in CHP100 cells (ES cell line). (E) Phosphorylation of ERBBs was examined by western blotting using phospho-specific antibodies. LP9, non-tumorigenic mesothelial cell line. (F) EGFR was immunoprecipitated from JN-DSRCT-1 cells and then western blot analysis was conducted to identify other ERBB family RTKs that are associated with it.

The results presented in Fig. 1 indicate that transcriptional signatures of activated kinase signaling pathways are present in DSRCT. To determine which of these pathways are activated at the proteomic level, we profiled the phosphorylation state of 49 RTKs using phospho-proteomic arrays in the five DSRCT cell lines and five DSRCT patient tumors (Fig. 4A-D). Protein phosphorylation was quantitated by densitometry and is shown in Fig. 4B and D. We found that members of the ERBB family RTKs were phosphorylated in the five DSRCT cell lines, with phosphorylation of EGFR, ERBB4 and ERBB2 consistently high in all cell lines (Fig. 4A). Note that due to the use of different primary antibodies on the array, the absolute levels of phosphorylation of different ERBB RTKs cannot be accurately compared to each other in a given sample. Only phosphorylation of EGFR was consistently observed in multiple DSRCT cell lines and tumors (Fig. 4B,D). By comparison, EGFR phosphorylation was much lower in ES and SS cells (CHP100 and SYO-1, respectively; Fig. S2A). To validate these data, we performed immunoblotting using phospho-specific and total anti-ERBB family antibodies, using the untransformed mesothelial cell line LP9 (Connell and Rheinwald, 1983) as a control (Fig. 4E). Immunoblots were quantitated, and the data are shown in Fig. S2B. Phosphorylated EGFR, ERBB2 and ERBB4 were detected in all DSRCT cell lines (Fig. 4E) at levels higher than in the control LP9 cell line (Fig. S2B). Consistent with the low signal in the RTK proteomic arrays, phosphorylated ERBB3 was extremely low (Fig. 4E). To determine whether these activated ERBB RTKs interacted with each other, we immunoprecipitated EGFR from JN-DSRCT-1 cell extracts and then immunoblotted for each ERBB RTK (Fig. 4F). We were able to clearly detect ERBB2 and ERBB4 in anti-EGFR immunoprecipitates, suggesting that these three RTKs interacted or colocalized. There was also a very weak band on the ERBB3 western blot, indicating that there may also be some interaction between EGFR and ERBB3.

**Fig. 5.**
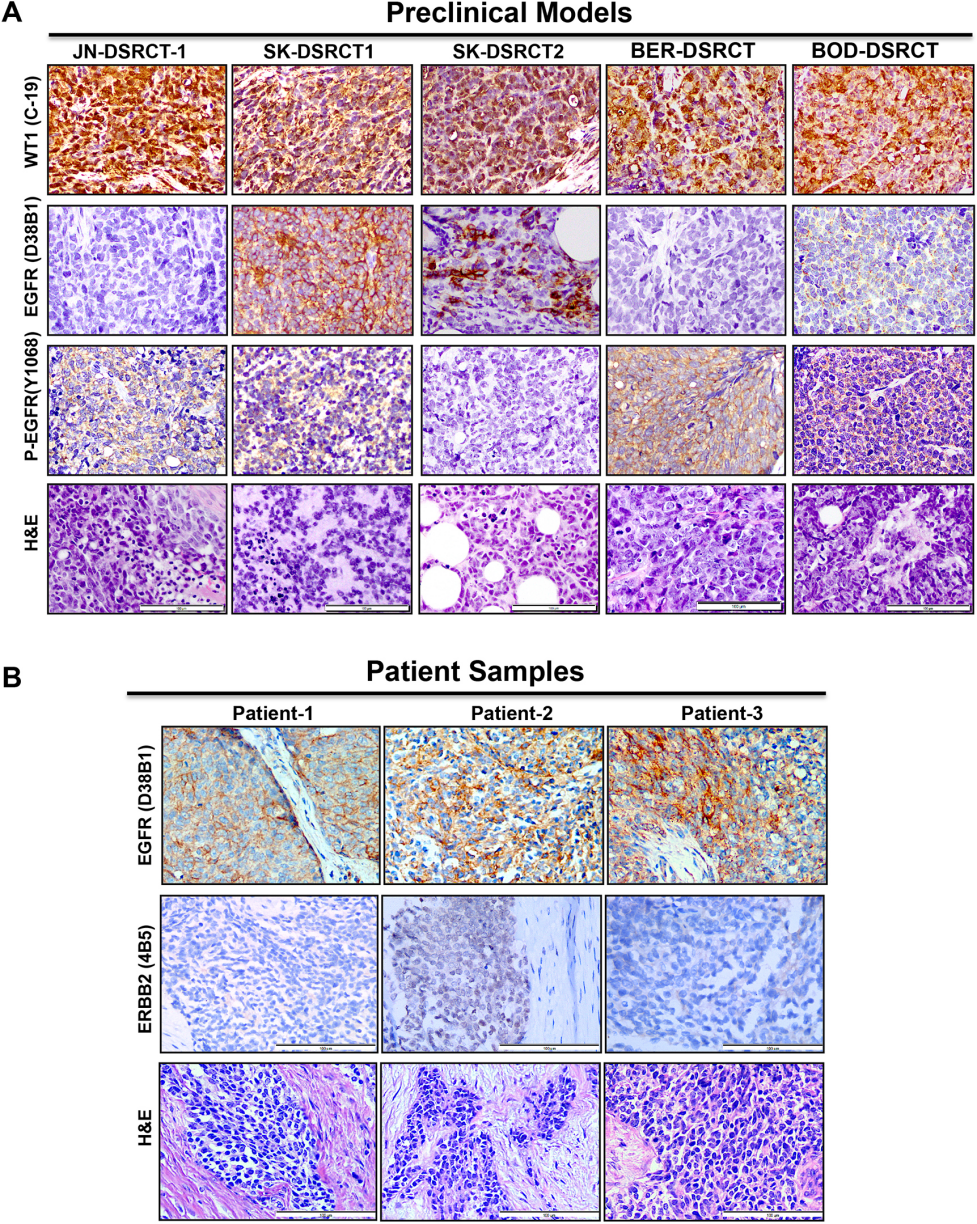
**Detection of WT1, EGFR, phospho-EGFR and ERBB2 by immunohistochemistry.** (A) Expression of WT1, EGFR and phospho-EGFR in paraffin-embedded xenograft tumors or cell pellet (SK-DSRCT1). Hematoxylin and Eosin (H&E) staining of corresponding slides is shown in the bottom row. (B) Expression of EGFR and ERBB2 in DSRCT patient samples. H&E staining of corresponding slides is shown in the bottom row. Representative photomicrographs are shown. Scale bars: 100 µm.

We performed immunohistochemistry (IHC) on paraffin-embedded DSRCT cell line xenografts or cell pellets (SK-DSRCT1) to confirm the presence of WT1 using an antibody raised against the C-terminus of WT1, and to explore expression of EGFR and phosphorylated EGFR, given that this ERBB RTK was the most consistently activated in cell lines and tumors tested. Strong nuclear staining for the WT1 C-terminus was detected in all samples probed (Fig. 5A), most likely representing EWS-WT1 in these novel DSRCT models, as a recent transcriptome profiling study has confirmed a lack of native *WT1* transcripts in DSRCT (Hingorani et al., 2020). EGFR was detected at varying levels in the different xenografted DSRCT cell lines or formalin-fixed paraffin-embedded SK-DSRCT1 cells. The weak EGFR staining of JN-DSRCT-1 xenograft tissue is consistent with the weak EGFR IHC staining that we previously observed in a JN-DSRCT-1 cell block (Saito et al., 2012). However, phosphorylated EGFR was detected in all samples, consistent with our *in vitro* studies (Fig. 5A). We next examined the level of EGFR and ERBB2 in all three patient samples by IHC. EGFR expression was detected in the three samples; however, ERBB2 was only modestly expressed (Fig. 5B).

### The ERBB pathway regulates growth of DSRCT cells

**Fig. 6.**
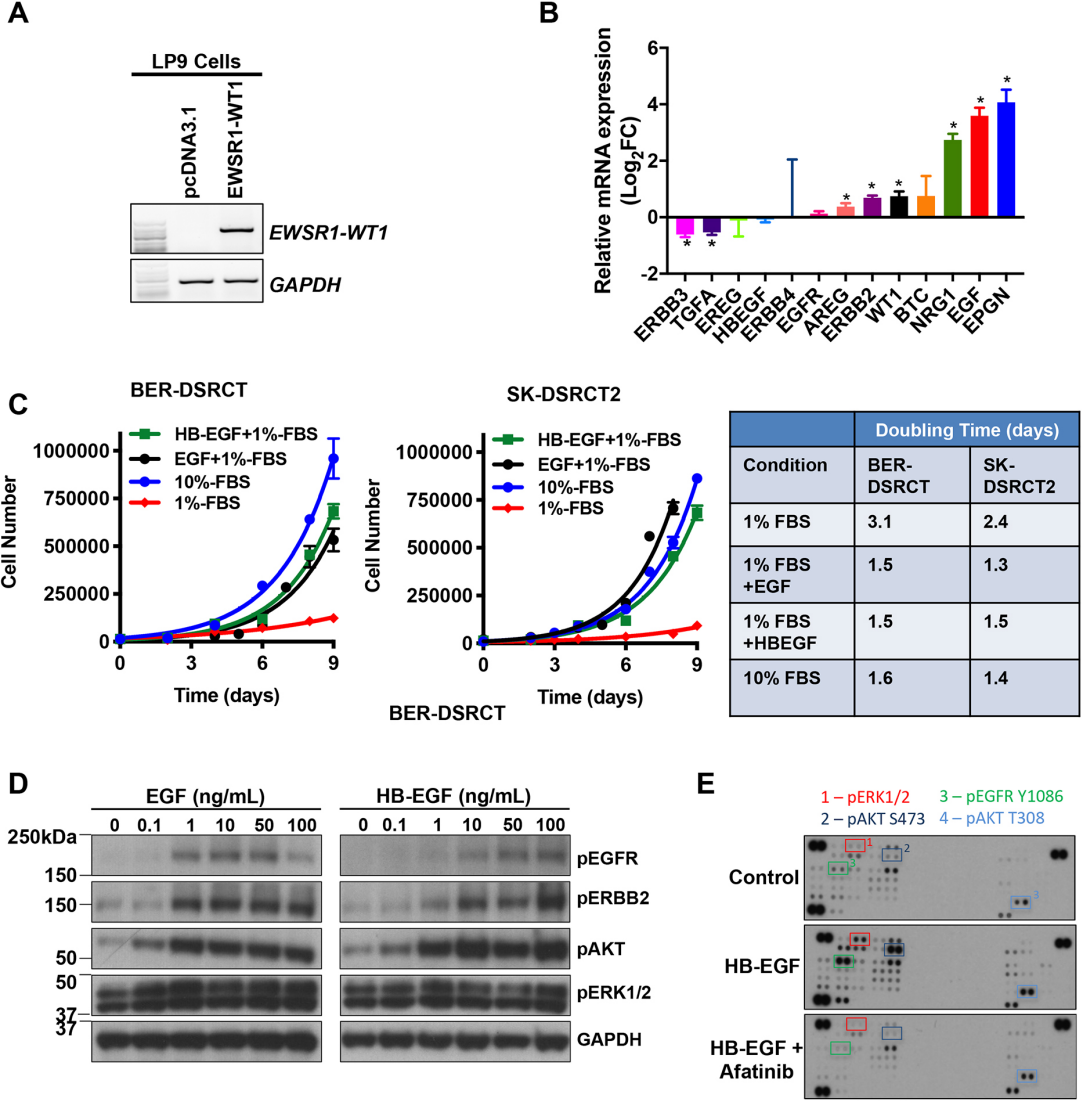
**ERBB pathway genes are altered by expression of EWS-WT1 and regulate growth of DSRCT cell lines.** (A,B) A cDNA encoding EWS-WT1 or empty plasmid (pcDNA 3.1) was expressed in LP9 cells (A) and then expression of known ERBB receptors and ligands was determined by qPCR (B). Expression of *EWSR1-WT1* was confirmed by RT-PCR. mRNA levels in LP9-EWS-WT1 cells are expressed relative to that in LP9-pcDNA3.1 (empty plasmid). *P<0.05 (unpaired, two-tailed Student’s *t*-test). (C) EGFR ligands are sufficient to stimulate growth of DSRCT cell lines. Results represent the mean±s.d. of two independent experiments. Growth data were fitted to an exponential growth equation using GraphPad Prism 7 software and the doubling time is shown in the right panel. (D) BOD-DSRCT cells were serum starved for 24 h and then stimulated with the indicated concentrations of EGF or HB-EGF for 15 min. Western blotting was then performed for the phosphorylated proteins shown or GAPDH. (E) BER-DSRCT cells were pretreated with DMSO or 0.25 μM afatinib for 30 min and then stimulated with 100 ng/ml HB-EGF for 15 min. Phosphokinase arrays were then used to assess the phosphorylation state of selected signaling proteins. (E) Representative images of phosphokinase arrays. Arrays were quantitated by densitometry, and the relative changes in phosphorylation above DMSO-treated control cells are shown in Fig. S3.

To explore the relationship between expression of EWS-WT1 and the ERBB pathway in an isogenic system, we asked whether expression of EWS-WT1 regulated components of the ERBB signaling axis. A cDNA encoding *EWSR1-WT1* was stably transfected into LP9 cells and then expression of the mRNAs for ERBB RTKs and ligands was determined in LP9-empty vector and LP9-EWS-WT1 cells using a custom qPCR array (Table S3). Expression of *EWSR1-WT1* in LP9 cells is shown in Fig. 6A. EWS-WT1 expression in LP9 cells caused a significant increase in mRNAs for several ERBB pathway ligand genes, including *NRG1*, *EGF*, *AREG* and *EPGN* (Fig. 6B). There was no change in *EGFR* mRNA, whereas *ERBB2* mRNA increased after EWS-WT1 expression, and there was a significant decrease in *ERBB3* mRNA (Fig. 6B). These results led us to hypothesize that ligands of the ERBB pathway can drive growth of DSRCT cells.

To address this, we treated two DSRCT cell lines with EGF or HB-EGF and examined growth and signaling pathways for activation. Chronic treatment of cells with EGF or HB-EGF was sufficient to drive a significant increase in cell growth, achieving the same level as seen in cells grown in 10% fetal bovine serum (FBS) (Fig. 6C). Treatment of DSRCT cell lines with EGF or HB-EGF stimulated phosphorylation of EGFR and ERBB2 in a dose-dependent manner (Fig. 6D). Concomitantly, the downstream effectors AKT and ERK1/2 (also known as MAPK3/1) were activated, as expected (Fig. 6D).

For a more comprehensive view of activated pathways in DSRCT cells, we employed a phospho-kinase array that contains antibodies that recognize 43 cytoplasmic phospho-proteins known to be activated by growth factors, to profile the signaling pathways activated by treatment with an ERBB ligand in the absence or presence of the pan-ERBB inhibitor afatinib (Fig. 6E). The relative protein phosphorylation was quantitated by densitometry and is presented in Fig. S3. Stimulation of BER-DSRCT cells with HB-EGF resulted in activation of multiple effector kinases, and this effect was mitigated by afatinib treatment.

**Fig. 7.**
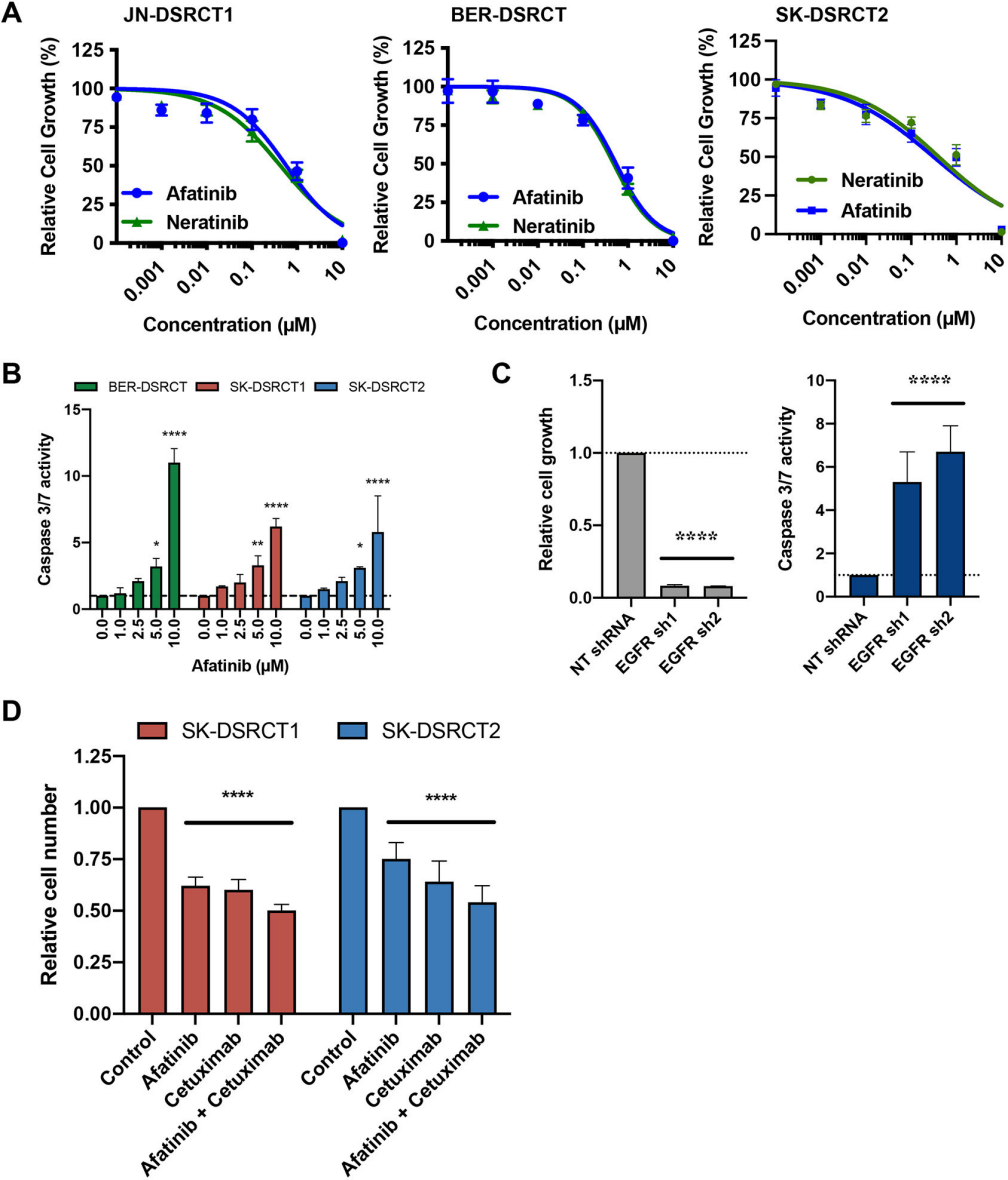
**EGFR antagonists inhibit growth of novel DSRCT cell lines.** (A,B) Cells were treated with increasing doses of afatinib or neratinib and then either viability (A) or caspase 3/7 activity (B) was measured. Results represent the mean±s.d. of three (A) or two (B) independent experiments in which each condition was assayed in triplicate (A) or duplicate (B) determinations. (C) BER-DSRCT cells were infected with lentivirus harboring shRNAs (scrambled control or targeting *EGFR*) and then the relative number of cells (left) or caspase 3/7 activity (right) was determined. Results represent the mean±s.d. of two experiments in which there were three replicates of each condition. (D) Cells were treated with 1 μM afatinib, 100 ng/ml cetuximab, or 1 μM afatinib+100 ng/ml cetuximab for 48 h.****P<0.0001, **P<0.001, *P<0.05, compared to vehicle-treated or non-targeting (NT) shRNA control (unpaired, one-tailed Student’s *t*-test).

To validate EGFR as a target for therapy in DSRCT, we examined the effect of pharmacological and genetic antagonists of EGFR on growth and survival. For these studies, we used afatinib and neratinib, two pan-ERBB inhibitors that have been shown to be active *in vitro* and *in vivo*. Treatment of JN-DSRCT-1 (Fig. 7A, left), BER-DSRCT (Fig. 7A, middle) and SK-DSRCT2 (Fig. 7A, right) cells with afatinib or neratinib inhibited growth of the cell lines in a dose-dependent manner, with half inhibitory concentration (IC_50_) values of 0.3-0.6 µM (Fig. S4). Growth of three cell lines derived from ES (CHP100, TC71) or SS (SYO-1) and LP9 cells was less sensitive to inhibition by afatinib (Fig. S4). The inhibition of growth of DSRCT cell lines by afatinib was accompanied by an increase in apoptosis, as treatment of cells with afatinib stimulated caspase 3/7 activity in the three DSRCT cell lines tested (Fig. 7B). Consistent with these findings, genetic inhibition of *EGFR* using two unique lentiviral delivered short hairpin RNAs (shRNA) in BER-DSRCT cells resulted in significant reduction in cell number relative to a scrambled shRNA control (Fig. 7C, left). The reduction in cell number coincided with a large increase in apoptosis relative to control shRNAs as measured by caspase 3/7 activity (Fig. 7C, right). Treatment of SK-DSRCT1 and SK-DSRCT2 for 48 h with afatinib, cetuximab or a combination of both also resulted in significantly reduced cell number (Fig. 7D). The consistency of these results confirms the sensitivity of these tumors to EGFR inhibition.

**Fig. 8.**
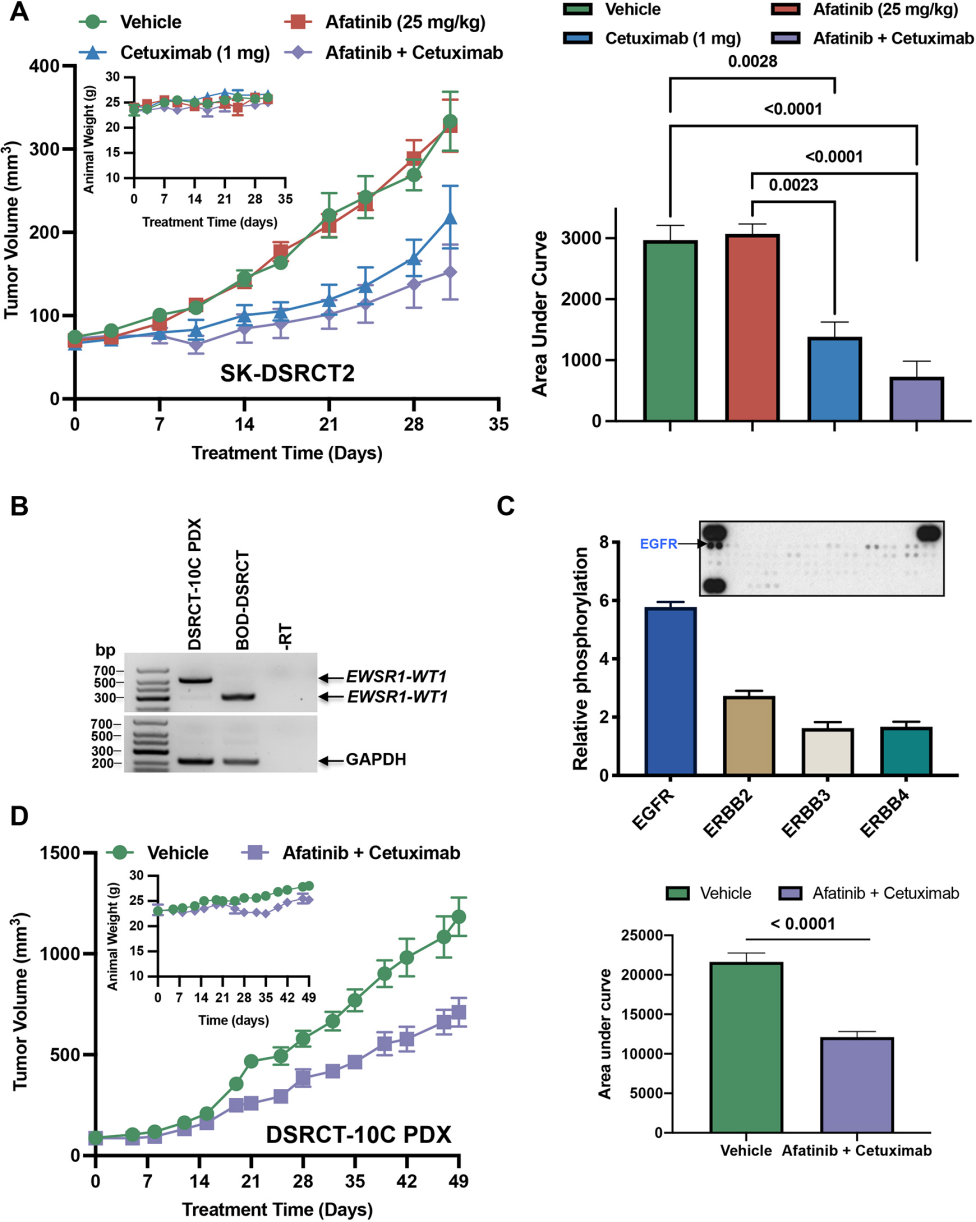
**EGFR antagonists inhibit growth of novel DSRCT xenograft tumors.** (A-D) SK-DSRCT2 cells (A) or DSRCT-10Cpdx tumors (B-D) were implanted subcutaneously into the flank of immunocompromised mice and treatment began when tumors reached ∼100 mm^3^. Mice were treated with vehicle, afatinib (25 mg/kg, QD, 5 days/week), cetuximab (1 mg, BIW), or a combination of cetuximab and afatinib. (A) Tumor volume measurements of SK-DSRCT2 xenografts with animal weight shown in the inset. Treatment was initiated 53 days after implantation. Area-under-the-curve (AUC) analysis is shown on the right. (B) Expression of *EWRS1-WT1* fusion was confirmed in the DSRCT-10Cpdx tumor by RT-PCR. (C) The phosphorylation level of RTKs was examined using an RTK array. The array was quantitated by densitometry, and relative levels of each phosphorylated ERBB family member are shown in the accompanying graph. (D) DSRCT-10Cpdx tumor volume is shown, demonstrating efficacy of combination therapy with afatinib (25 mg/kg, QD, 5 days/week) and cetuximab (1 mg, BIW) with animal weight shown in the inset. No treatment caused any significant change in animal weight. Treatment was initiated 20 days after implantation. AUC analysis is shown on the right. Groups were compared by two-way ANOVA with Tukey's multiple comparisons test. For the DSRCT-10Cpdx studies, there were four animals per group; for the SK-DSRCT2 xenograft studies, there were five animals per group. All measurements are mean±s.e.m.

To begin to translate these findings to DSRCT patients, we examined the efficacy of targeting EGFR in xenograft models. Cells (BER-DSRCT, SK-DSRCT2) or PDX (DSRCT-10Cpdx) tissues were implanted into the subcutaneous flank of immunocompromised mice and animals were treated with vehicle [once per day (QD)], afatinib (25 mg/kg QD), cetuximab [1 mg two times per week (BIW)], or a combination of cetuximab (1 mg BIW) and afatinib (25 mg/kg QD). The tumor volumes over the course of treatment are shown in Fig. 8 (SK-DSRCT2, DSRCT-10Cpdx) and Fig. S5A (BER-DSRCT). We used area-under-the-curve (AUC) analysis to compare the effect of treatment between groups, as this analysis takes into account the magnitude and duration of the treatment effect. Cetuximab treatment reduced the growth of SK-DSRCT2 (Fig. 8A) and BER-DSRCT (Fig. S5B) tumors significantly. However, afatinib monotherapy did not cause a statistically significant reduction in growth of any of the cell line xenograft tumors (Fig. 8A; Fig. S5A), even though there was a modest reduction in growth of BER-DSRCT xenograft tumors (Fig. S5B). We extended these studies to include a DSRCT PDX model that we developed (DSRCT-10Cpdx). We confirmed that DSRCT-10Cpdx expresses the *EWSR1-WT1* fusion (Fig. 8B). Similar to the cell lines and patient samples shown in Fig. 4, the patient sample from which the DSRCT-10Cpdx model was derived had elevated levels of phosphorylated EGFR (Fig. 8C). The combination of afatinib and cetuximab significantly reduced growth of this PDX model (Fig. 8D).

## DISCUSSION

The novel DSRCT models that we describe here represent an invaluable tool to the research community to investigate this aggressive sarcoma of adolescents and young adults. By analyzing transcriptomic and proteomic profiling of DSRCT tumors and cell lines, we find ERBB signaling pathways to be activated in DSRCT relative to other sarcomas. Further, we show that EGFR is a determinant of growth in these tumors and can be exploited for therapy.

Our study focused primarily on developing and characterizing new DSRCT preclinical models, and examined kinases as a potential therapeutic target. Determining precisely how the ERBB and other kinase signaling pathways are dysregulated and co-opted by the EWS-WT1 chimeric transcription factor to drive tumor progression remains an open question and one that we can begin to investigate more effectively with these new DSRCT models. EWS-WT1 presides over an aberrant gene expression program that drives tumorigenesis. In an effort to identify genes regulated by this oncogenic transcription factor, early studies reported that ectopic expression of EWS-WT1 resulted in upregulation of platelet-derived growth factor-alpha (*PDGFA*) mRNA (Lee et al., 1997). The *PDGFA* gene promoter includes several binding sites for WT1, suggesting that this gene is likely transcriptionally regulated by EWS-WT1 (Lee et al., 1997). Subsequent studies by other groups have shown that EWS-WT1 can regulate expression of *EGR1* (Liu et al., 2000) and IGF1 (Karnieli et al., 1996). However, given the lack of DSRCT models, it was not possible to perform preclinical studies to evaluate the therapeutic relevance of those observations. More recently, we showed that *NTRK3* was also transcriptionally regulated by EWS-WT1 and that NTRK3 can be exploited as a potential therapeutic target for this disease (Ogura et al., 2021).

Using the new DSRCT cell lines, we have approached the challenge of developing targeted therapy for DSRCT in an unbiased approach, asking which known targetable growth pathways might be dysregulated and contributing to DSRCT proliferation. We found that EGFR is consistently phosphorylated in DSRCT cell lines, PDX and patient tumors. Moreover, expression of the EWS-WT1 oncoprotein in LP9 cells dysregulated the ERBB pathway, increasing the expression of several ligands, including EGF, amphiregulin, epiregulin and neuregulin 1. It is likely that the increased level of these ligands is responsible for the increased activation of EGFR (and other ERBB receptors) observed, as we also demonstrate that treating DSRCT cells with EGF is sufficient to increase proliferation.

Binding of ligands to EGFR leads to homodimerization or heterodimerization with other ERBB family members, depending on the ligand (Uribe et al., 2021). Once activated, EGFR is able to bind directly to adaptor or effector molecules, initiating a signaling cascade that results in activation of potent growth signaling pathways (e.g. RAS-MAPK, PI3K-AKT, STAT3, PLCγ, etc.). Here, we showed that the RAF and MEK pathways were transcriptionally upregulated in DSRCT tumors compared to other sarcomas and that treatment of DSRCT cells with EGF or HB-EGF caused only a small increase in ERK1/2 phosphorylation, probably due to high basal activation of the MAPK pathway by EWS-WT1. Afatinib treatment decreased HB-EGF-stimulated activation of ERK1/2 to below basal levels, raising the possibility that a significant proportion of MAPK pathway activation may be under the control of ERBBs. In contrast to the effect seen with ERK1/2, ligand treatment resulted in a robust stimulation of AKT phosphorylation. Future studies are needed to determine more precisely how the PI3K-AKT and other activated pathways [NTRK3 (Ogura et al., 2001), FGFR4 (Saito et al., 2012; Hingorani et al., 2020)] are co-opted by EWS-WT1 to promote DSRCT tumorigenesis. Overall, these results raise the possibility of targeting AKT or ERK1/2 in combination with RTK tyrosine kinase inhibitors or standard therapy to improve responses.

To begin to evaluate the potential clinical relevance of our findings, we explored the effect of inhibiting EGFR in cell lines and xenograft models. Both genetic and chemical perturbation of EGFR signaling resulted in reduced growth and increased apoptosis. Extending these studies to animal models, we found that cetuximab was the most effective anti-EGFR agent for reducing tumor burden in mice bearing DSRCT tumors. Although the reductions in tumor growth obtained when EGFR antagonists were administered *in vivo* were only partial, we believe that these findings are promising and should be further explored to determine whether different dosing or combination with chemotherapy or other targeted agents would offer additional benefits for patients. Given the poor response and limited overall survival of patients with DSRCT, our findings begin to provide new therapeutic options where they are desperately needed.

## MATERIALS AND METHODS

Cell culture media, antibiotics and phosphate-buffered saline (PBS) were prepared by the MSK Media Preparation Core Facility. FBS was procured from Atlanta Biologicals (Flowery Branch, GA, USA). HEK-293T cells were obtained from American Type Culture Collection (Manassas, VA, USA). The JN-DSRCT-1 cell line was a gift from Dr Jun Nishio (Fukuoka University, Fukuoka, Japan). The LP9 cell line is an untransformed, diploid, mesothelial cell line that was derived from a 26-year-old female ovarian cancer patient (Connell and Rheinwald, 1983) and was obtained from the NIGMS Human Genetic Cell Repository at the Coriell Institute For Medical Research (AG07086) (Camden, NJ, USA). Antibodies to all native and/or phosphorylated proteins (see Table S3 for details) used in western blotting studies except for WT1 were purchased from Cell Signaling Technology (Boston, MA, USA). The C-terminal anti-WT1 antibody (C-19) used for immunohistochemistry was purchased from Santa Cruz Biotechnology (Dallas, TX, USA). The anti-WT1 antibody used for western blotting was obtained from ThermoFisher Scientific (Waltham, MA, USA). All primary antibodies were used at a dilution of 1:1000 for western blotting. Small-molecule inhibitors were obtained from Selleckchem (Houston, TX). Promega's Apo-ONE Homogenous Caspase 3/7 Assay Kit, AlamarBlue, puromycin, geneticin, tissue culture plastic wares and all western blotting reagents were obtained from ThermoFisher Scientific. Protease inhibitor cocktail, RIPA buffer (10×) and all other chemicals not listed above were purchased from EMD-Millipore Sigma (St Louis, MO, USA). All oligonucleotides used for PCR assays were obtained from Integrated DNA Technologies (Coralville, IA, USA).

### Generation and growth of cell lines

Tissue samples were collected under an Institutional Review Board (IRB)-approved biospecimen collection protocol and informed consent was obtained from patients. The SK-DSRCT-1 cell line was generated from ascites fluid. Briefly, ascites fluid was centrifuged at 300 ***g*** for 5 min to pellet cells, and then pellets were washed twice with ice-cold PBS, centrifuged again and finally resuspended in Dulbecco's modified Eagle medium (DMEM):F12 (1:1 ratio) supplemented with 10% FBS and 1% antibiotics. The cell lines were considered established after growing for 20 continuous passages. The SK-DSRCT2 cell line was created from a surgically resected abdominal tumor, which was cut into small pieces with a scalpel in serum-free DMEM:F12 growth medium and then digested for 1 h with collagenase (2 mg/ml) in a final volume of 5 ml, at 37°C. The sample was vortexed every 5 min and then DME:F12+10% FBS medium was added to a final volume of 50 ml, centrifuged to pellet cells and then plated in DMEM:F12 growth medium supplemented with 10% FBS and 1% antibiotics. The sample was allowed to propagate over multiple generations, trypsinized when necessary to subculture, and, eventually, only single cells remained. Unless indicated otherwise, all cell lines were maintained in DMEM:F12 growth medium supplemented with 10% FBS and 1% antibiotics in a humidified incubator infused with 5% CO_2_ and subcultured when stock flasks reached ∼75% confluence at a 1:3 dilution. BER-DSRCT and BOD-DSRCT cell lines were derived from DSRCT patient-derived xenograft tissues as described previously (Markides et al., 2013). For determination of doubling time, cells were plated at a density of 25,000 (JN-DSRCT-1, SK-DSRCT1, BOD-DSRCT, BER-DSRCT) or 50,000 (SK-DSRCT2) cells per well in six-well plates and then counted every 24 h. Data points were fitted to an exponential growth equation using GraphPad Prism to determine the doubling time. Clonogenic assays were used to determine the plating efficiency of each cell line (Rafehi et al., 2011). Cells were plated at three densities in 6-mm dishes (1000, 2000 or 3000 cells per dish) in triplicates and grown for 2 weeks. Colonies were then stained with Crystal Violet and counted. Plating efficiency was determined as the number of colonies/number of cells plated for each density and then averaged. Only colonies of >100 cells were counted. For ligand-induced growth, BER-DSRCT and SK-DSRCT2 cells were plated in 12-well plates at a density of 10,000 cells/well, and, 24 h after plating (day 0), cells were counted and then placed in media containing 10% FBS, 1% FBS or 1% FBS supplemented with 100 ng/ml EGF or 100 ng/ml HB-EGF. Cells were counted every 24-48 h thereafter. For immunoprecipitation studies, cells were plated at a density of 5 million cells in 10-cm dishes. Two days later, cells were depleted of serum for 24 h before preparation of cell extracts. All cell lines were tested for mycoplasma every 3-4 months. Cell lines were profiled by MSK-IMPACT as described below. All cell lines used in this study were verified by PCR testing for the driver fusion genes each time a new vial was thawed.

### Spectral and DAPI karyotyping

Metaphase preparations were hybridized with spectral karyotyping (SKY) painting probe according to the manufacturer's recommendations (Applied Spectral Imaging) and a minimum of ten metaphases analyzed (Imataka and Arisaka, 2012; Schröck et al., 1996). Additionally, a minimum of 20 DAPI-banded metaphases was analyzed to better define the chromosomal breakpoints and intrachromosomal rearrangements. Karyotyping was conducted by the MSK Cytogenetics Core Facility and all metaphases were fully karyotyped.

#### Genomic characterization

Cell lines were profiled by the MSK-IMPACT platform, which is a large panel sequencing (NGS) assay that was used here to detect mutations and copy number alterations involving up to 468 cancer-associated genes (Cheng et al., 2015). As the corresponding patient-matched normal DNA was unavailable, single-nucleotide variants (SNVs) representing known COSMIC somatic mutations or truncating mutations in tumor suppressor genes and CNVs were tabulated.

### Viability and caspase 3/7 assays

For viability assays, cells were plated in clear-bottom, white 96-well plates at a density of 5000 cells per well and incubated with compounds for 96 h. The relative amount of viable cells was determined using AlamarBlue viability dye and fluorescence was measured using a SpectraMax M2 plate reader (excitation, 485 nm; emission, 530 nm) (Somwar et al., 2009). Data were analyzed by non-linear regression and curves fitted using GraphPad Prism software to generate IC_50_ values. For caspase 3/7 activity, cells were plated at a density of 30,000 cells/well directly into inhibitors in white, clear-bottom 96-well plates, grown for 48 h, and then caspase 3/7 enzymatic activity was determined using an Apo-ONE Homogenous Caspase 3/7 Assay Kit (Promega), following the manufacturer's instructions. All data are expressed relative to control values and are an average of two to five independent experiments in which each condition was assayed in triplicate determinations.

#### Plasmids, lentiviral generation and transfection

Bacteria stocks harboring the pLKO.1 MISSION lentiviral shRNA constructs were obtained from the MSK RNAi Core Facility. For packaging lentivirus, we used psPAX2 (Addgene) and VSV-G/pMD2 (Addgene). Viruses were generated and cells infected as we have described previously (Vojnic et al., 2019). LP9 cells were transfected with pCDNA3.1 harboring *EWSR1-WT1* fusion cDNA (cloned from the JN-DSRCT-1 cell line) or empty plasmid, and, 48 h later, cells were treated with 500 µg/ml geneticin to select stable cells. Stable cells were obtained after 3 weeks of antibiotic selection.

### Preparation of whole-cell extracts, immunoprecipitation and western blotting

Protein levels and phosphorylation state were detected by western blotting. For studies of ligand-dependent activation of signaling pathways, cells were deprived of serum for 24 h by growing in KSM-defined medium (serum-free medium, ThermoFisher Scientific) and then treated with ligands for 15 min in KSM-defined medium. Cells were lysed in 1× RIPA lysis buffer containing Halt protease and a phosphatase inhibitor cocktail according to the manufacturer's protocol (ThermoFisher Scientific). For immunoprecipitation studies, whole-cell extracts were prepared as described above and then EGFR was immunoprecipated from 300 µg total protein using an anti-EGFR antibody conjugated to agarose beads overnight. Immunoprecipitates were washed four times with ice-cold RIPA buffer containing protease and phosphatase inhibitors and then bound protein eluted with 100 µl 2× Laemmli sample buffer (LSB) with 5% reducing agent. Lysates or immunoprecipitates were denatured in 2× sample buffer at 55°C for 15 min, resolved on 4-12% NuPAGE gels (Invitrogen) and transferred onto polyvinylidene fluoride membranes. Membranes were blocked in 3% bovine serum albumin (BSA) in Tris-buffered saline supplemented with 0.1% Tween 20 (vol/vol) for 1 h at room temperature and probed with primary antibodies with specificity as outlined in Table S3. Proteins were separated on 8% NuPAGE gels for detection of WT1. Bound antibodies were detected with peroxidase-labeled goat antibody to mouse IgG or rabbit IgG (R&D Systems) and developed with enhanced chemiluminescence (ECL) western blotting detection reagent (GE Healthcare).

### Proteome profiling arrays

We used human proteome profiling arrays (R&D Systems) that contain duplicate validated controls and capture antibodies that can simultaneously detect the phosphorylation state of 43 human kinases (Proteome Profiler Human Phospho-Kinase Array Kit) or 49 RTKs (Proteome Profiler Human Phospho-RTK Array Kit). Five million cells were plated in 10-cm dishes and grown for 48 h. Cells were then deprived of serum overnight and detection of protein phosphorylation was carried out according to the manufacturer's instructions. In brief, the array membranes were blocked, incubated with 350 µg total cellular protein per array overnight at 4°C on a rocking platform, washed and incubated with antibodies. Captured phosphorylated proteins were detected by ECL and imaged on X-ray films. The pixel densities of duplicate spots were measured using ImageJ software (http://imagej.nih.gov/ij/), and expressed relative to the pixel density of the positive control on each array. This controls for any differences in exposure time. The levels of phosphorylation in DSRCT cells were then compared to the corresponding levels in CHP100 cells. Different capture antibodies were used for each specific RTK; therefore, it is not accurate to compare the phosphorylated levels of different RTKs. For the phosphokinase arrays, after normalizing each spot to the positive control, each value in the treated cells is expressed relative to the dimethyl sulfoxide (DMSO) control. The coordinates of the array to identify specific RTKs are provided in Table S3.

#### Detection of *EWSR1-WT1* fusion and qPCR for *WT1* and ERBB pathway gene expression

Total RNA was extracted using a Qiagen RNA mini kit and cDNAs were synthesized using SuperScript IV VILO (ThermoFisher Scientific) according to the manufacturers’ instructions. The *EWSR1-WT1* fusion was detected by RT-PCR using 5′-CTATTCCTCTACACAGCCGACT-3′ (forward, *EWSR1* exon 7) and 5′-CTGTATGTCTCCTTTGGTGTCT-3′ (reverse, *WT1* exon 8) and the following conditions in a PCR thermocycler: initial denaturation (95°C, 5 min), followed by 38 cycles of denaturation (95°C, 30 s), annealing (60°C, 30 s) and extension (72°C, 1 min); the reaction was finished after the final extension step (72°C, 5 min). For qPCR expression analysis, TaqMan assays were used and these are detailed in Table S3.

#### Histology and IHC

Histology and IHC were performed as previously described (Liu et al., 2015). Briefly, xenograft tissues were collected, fixed in 4% buffered formalin-saline at room temperature for 24 h, embedded in paraffin blocks, and then sections of 4 µM thickness were mounted on glass slides. For IHC assays, slides were immersed in 3% H_2_O_2_ for 5 min, washed, incubated for 15 min in 5% BSA to block non-specific binding sites and finally incubated in primary antibodies overnight at 4°C. The slides were washed the next day and then incubated with biotinylated anti-rabbit secondary antibody using a DAB kit (Dako) according to the manufacturer's protocol. Slides were counterstained with Hematoxylin. An anti-WT1 antibody that was raised against a peptide mapping at the C-terminal region of WT1 (C-19, Santa Cruz Biotechnology) was used. EGFR and phospho-EGFR IHC were conducted with anti-EGFR (D38B1) and anti-phospho-EGFR (Y1068) antibodies, respectively, obtained from Cell Signaling Technology. IHC staining of patient samples was performed with the VENTANA BenchMark ULTRA IHC system. ERBB2 IHC was conducted using an anti-ERBB2 antibody (4B5) purchased from Roche.

### Establishment of patient-derived cell lines and xenografts, and efficacy studies

Patient-derived cell lines and xenografts were developed under IRB-approved biomarker specimen protocols (06-107, 14-091) and all patients consented to collection and use of tumor material. Mice were cared for in accordance with guidelines approved by the Memorial Sloan Kettering Cancer Center Institutional Animal Care and Use Committee, and Research Animal Resource Center. Animals were monitored daily. Ascites and tumor samples were collected from routine procedures in sterile containers. Heparin was added to fluids at the time of collection and cells were collected by centrifugation (300 ***g***, 5 min), washed once with ice-cold PBS and then either resuspended in growth medium (DMEM:F12+10% FBS+1% antibiotic/antimycotic solution) or prepared as described next for solid tumors for implantation into mice. Fresh tumor samples were cleaned and then minced, mixed with Matrigel and implanted into a subcutaneous flank of 6-week-old female *NOD*/*SCID* gamma (NSG; The Jackson Laboratory, Bar Harbor, ME, USA) mice to generate xenografts (Mattar et al., 2018). For cell line xenografts, 10 million cells were mixed with Matrigel (1:1) and injected subcutaneously into a single flank of female NSG mice. Once tumors reached ∼100 mm^3^ volume, mice were randomized into groups of four (BER vehicle, DSRCT-10Cpdx afatinib+cetuximab) or five mice to achieve similar average starting tumor volume and weight across groups, and tumor-bearing animals were treated with vehicle, afatinib (25 mg/kg QD), cetuximab (1 mg BIW), afatinib (25 mg/kg QD)+cetuximab (1 mg BIW) when tumors reached ∼100 mm^3^ volume. Afatinib was resuspended in 0.5% methylcellulose and 0.4% Tween 80, and was given by oral gavage. Cetuximab was administered via intraperitoneal injection. Tumor size and body weight were measured twice weekly and tumor volume was calculated using the formula: length×width^2^×0.52.

### Microarray expression analysis

We mined legacy gene expression microarray data for 137 sarcoma samples (28 DSRCTs, 28 ESs, 23 fusion-positive ARMSs, 46 SSs and 12 ASPSs) that were generated using Affymetrix U133A arrays and used in previous publications (Tsuda et al., 2007; Filion et al., 2009; Kobos et al., 2013). The raw data are available at http://cbio.mskcc.org/public/sarcoma_array_data/filion2009.html. Data were Robust Multichip Average (RMA)-normalized and then linear regression analysis was performed using limma R package (Ritchie et al., 2015). GSVA was conducted with GSVA R package (Hanzelmann et al., 2013), and GSEA was performed using Java-based GSEA software (Hanzelmann et al., 2013). The GSVA computes enrichment scores for predefined gene sets (oncogenic signatures) for each sample separately, allowing for more flexible comparison of multiple groups and assessing of intra-group variability. The average scores in each group were then compared between sarcoma types using linear regression in limma (linear models for microarray data) (Ritchie et al., 2015). Exploratory analysis was performed using Rtsne and pheatmap packages. GSEA analysis was run using ‘gene_set’ permutation type against the oncogenic signatures gene sets database. pheatmap, ggplot2 and clusterProfiler R packages were used for data visualization (Yu et al., 2012).

### Statistical analysis

There were two to five replicates of each condition and all experiments were repeated two to four times. All data were plotted and analyzed using GraphPad Prism software. Viability data were analyzed by non-linear regression, and estimated IC_50_ values are given with their respective 95% confidence interval. Data are shown as mean±s.d. or s.e.m. in graphs. For animal studies, there were four or five mice in each group, and AUC analysis was used to compare the average tumor volume between groups. Briefly, AUC values and their standard errors were computed as an estimation of a surface area between baseline values (mean value of the tumor volumes at the beginning of the treatment) and growth curves for vehicle and each treatment conditions. Treatment response was compared to the vehicle group using two-way ANOVA with Tukey's multiple comparisons test. One-tailed Student's *t*-test (unpaired) was used to compare caspase activity or protein phosphorylation.

## Supplementary Material

Supplementary information
