# Supplementary material for: Novel patient-derived models of desmoplastic small round cell tumor confirm a targetable dependency on ERBB signaling
Source: Dis Model Mech. 2022 Jan 27;15(1):dmm047621. doi: 10.1242/dmm.047621 (PMC8807576; doi:10.1242/dmm.047621)
Supplement: Supplementary information [file dmm-15-047621-s1.pdf]

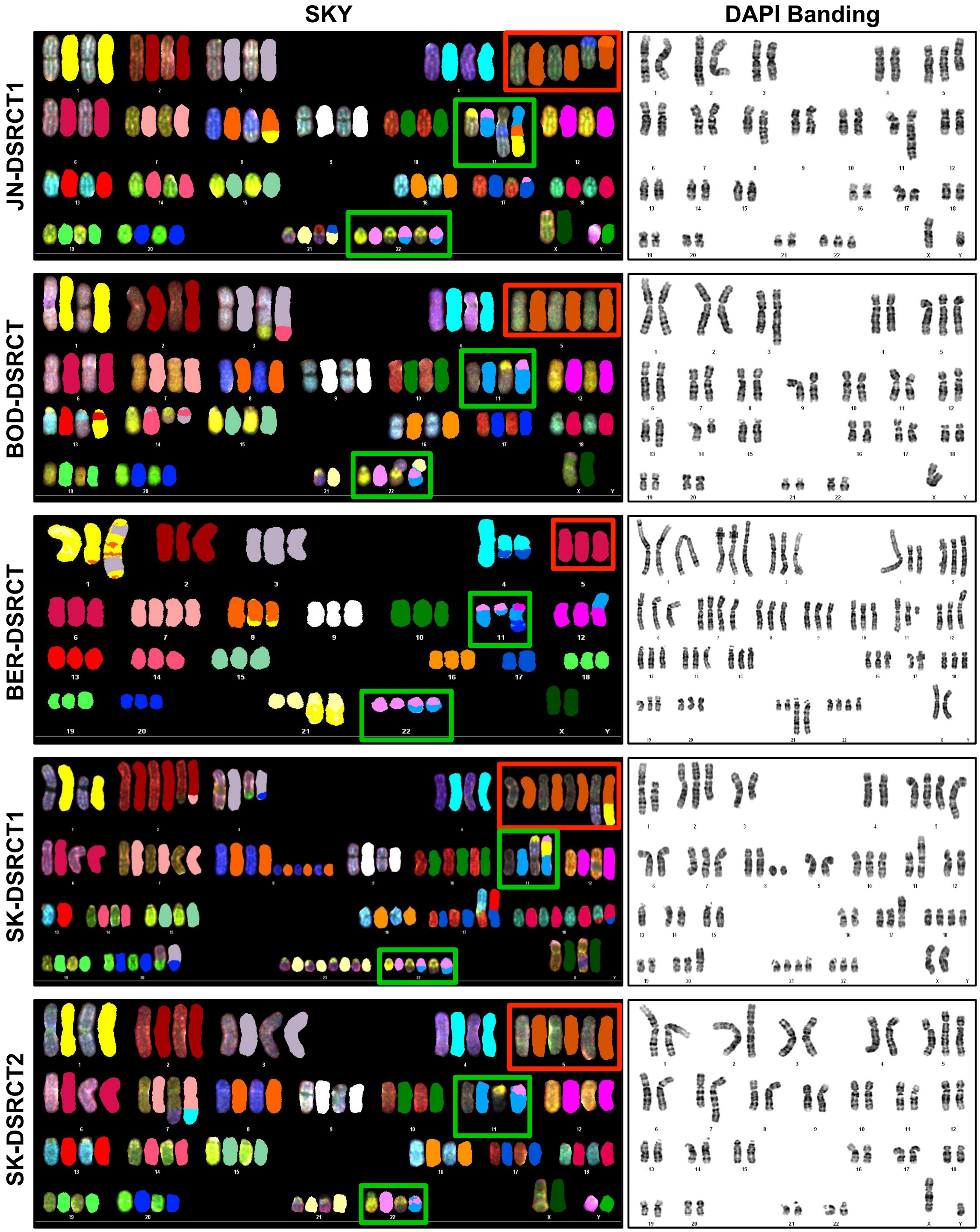

Fig. S1. Spectral (left) or DAPI-banded (right) karyotypes of all DSRCT cell lines. The translocation between chromosomes 11 and 22, resulting in the oncogenic chimeric transcription factor *EWSR1-WT1* is shown in the green box. Chr5 polysomy is shown in the red box.

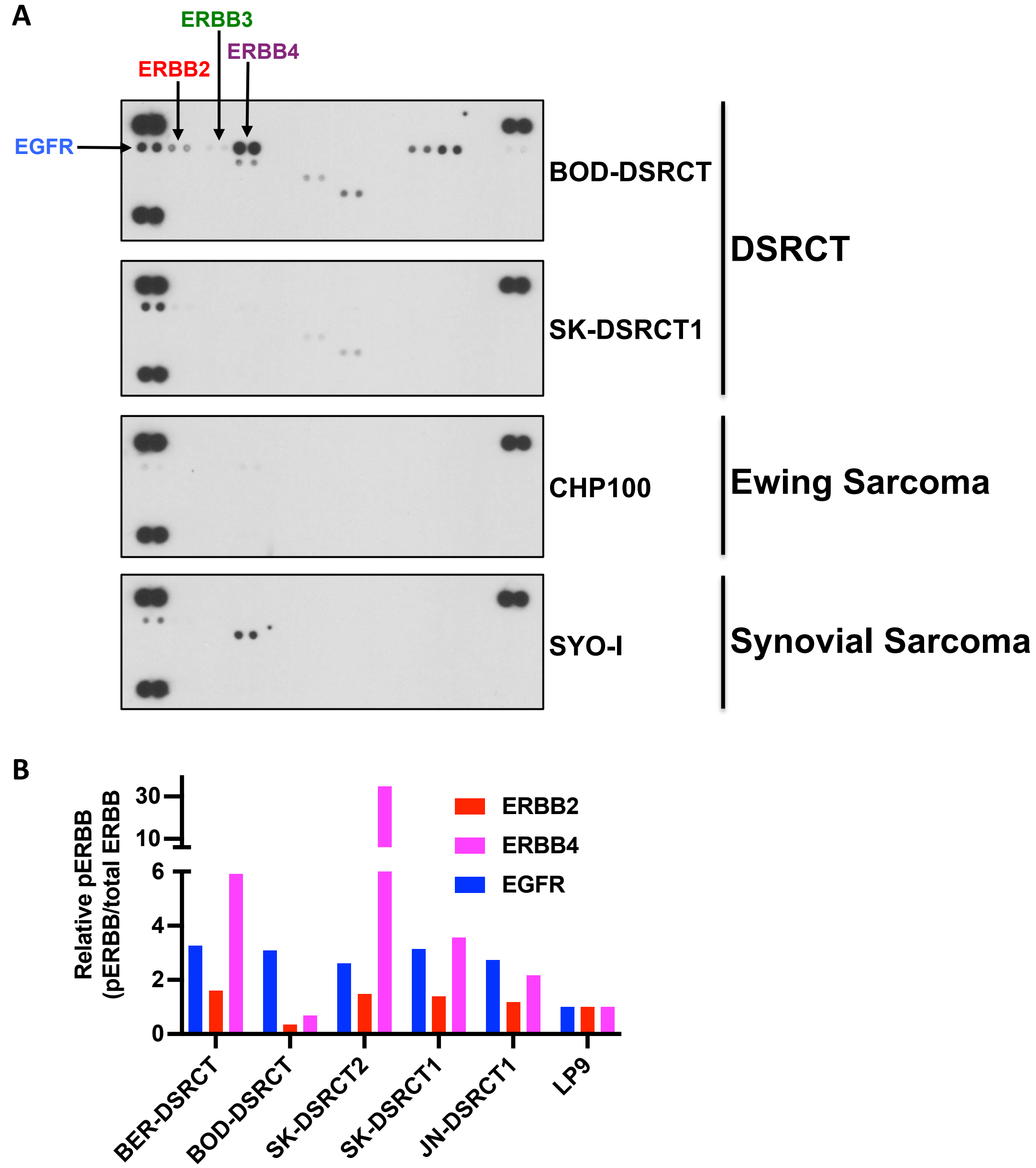

Fig. S2. EGFR activity is more prevalent in DSRCT cells than LP9 cells and other sarcomas. A. DSRCT, Ewing and synovial sarcoma cell lines were profiled for activated RTKs using phospho-RTK arrays. See Table S3 for coordinates to match the spots on the array to the RTK identity. B. Cell extracts were prepared from DSRCT and LP9 cells and then probed for phosphorylated or total EGFR, ERBB2, ERBB3 and ERBB4 by Western blotting. Representative immunoblots depicted in **Figure 4E** was quantitated by densitometry. The levels of phosphorylated proteins are expressed related to the level of total protein in each cell line and then normalized to LP9 cells. There was no detectable ERBB3 phosphorylation in most cell lines and therefore this was not quantitated. All cells were deprived of serum for 24 hours prior to preparation of cell extract.

Disease Models & Mechanisms • Supplementary information

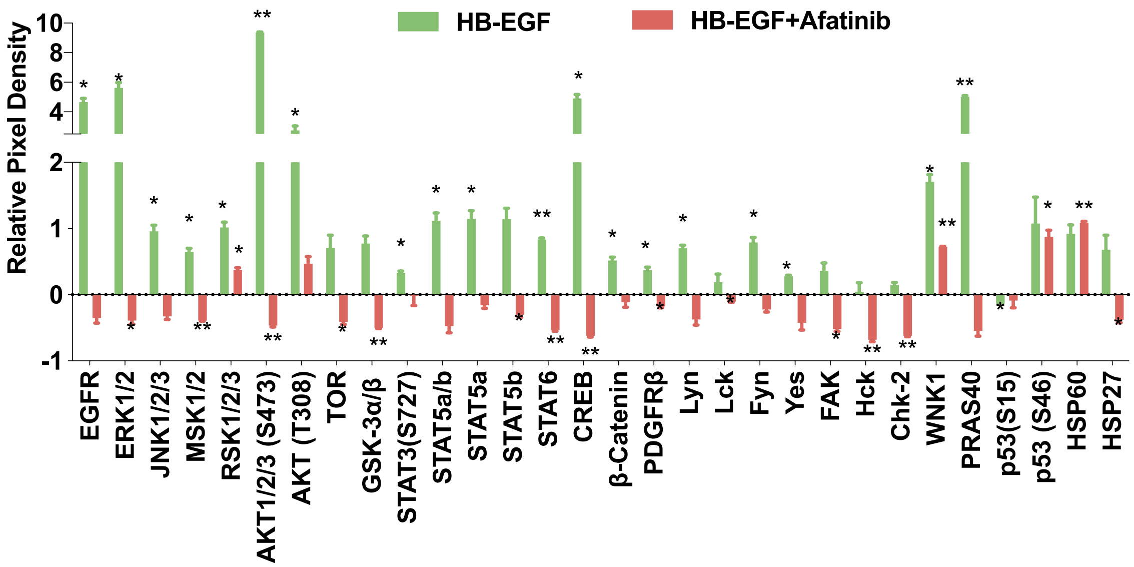

Fig. S3. Phospho-kinase arrays shown in Figure 6E were quantitated by densitometry and the relative change in phosphorylation above DMSO-treated control cells are shown. * P < 0.05, ** p < 0.01.

Disease Models & Mechanisms • Supplementary information

Cell Line	Tumor Type	IC_{50} For Growth Inhibition by Afatinib (µM)
JN-DSRCT1	DSRCT	0.6 (0.3-1.0)
BER-DSRCT	DSRCT	0.5 (0.3-0.8)
SK-DSRCT2	DSRCT	0.3 (0.2-0.5)
LP9	Non-tumor	5.0 (1.6-15)
CHP100	Ewing sarcoma	2.6 (1.5-4.3)
TC71	Ewing sarcoma	4.8 (2.3-9.7)
SYO1	Synovial sarcoma	4.9 (2.2-11)

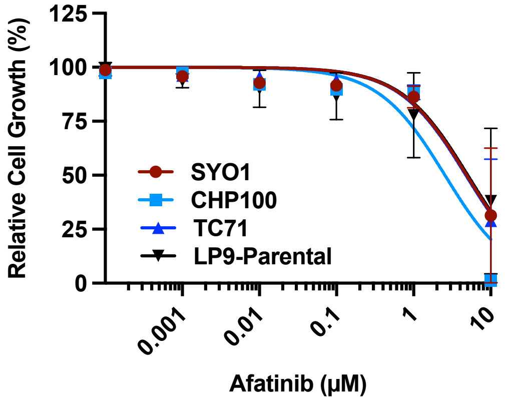

Fig. S4. DSRCT cells are more sensitive to EGFR inhibitors than other sarcomas. Cells were treated with afatinib for 96 h and then viability determined. Left: data was analysed by non-linear regression and curves fitted to generate IC_{50} values and the corresponding 95% confidence intervals (show in brackets). Right: growth curves showing sensitivity of non-DSRCT cell lines to afatinib. Results represent the mean ± s.d of 2-3 independent experiments in which each condition was assayed in triplicate determination. Analysis was performed using Graphpad Prism software.

Disease Models & Mechanisms • Supplementary information

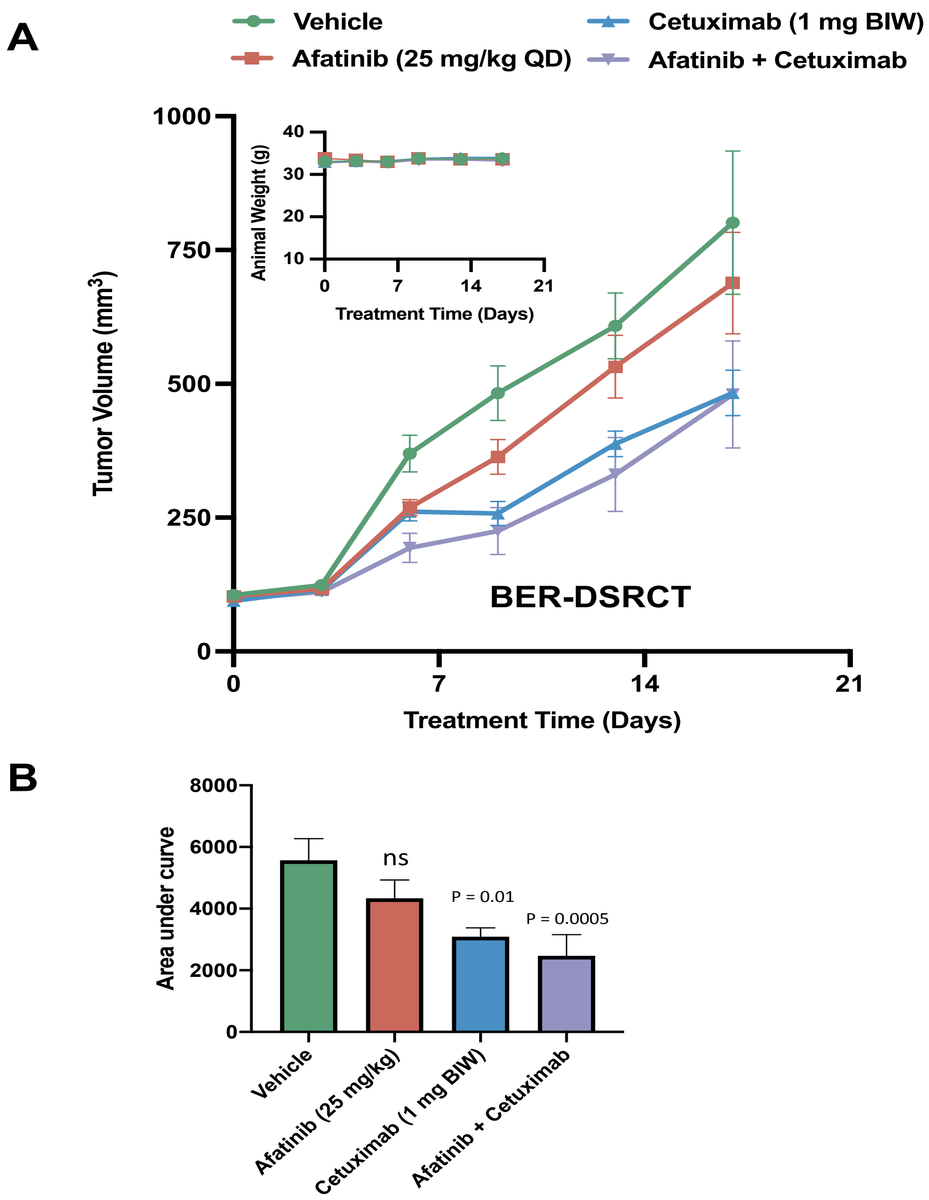

Fig. S5. BER-DSRCT cells were implanted subcutaneously into the flank of immunocompromised mice and treatment began when tumors reached approximately 100 mm^3. Mice were treated with vehicle, afatinib (25 mg/kg, QD, 5 days/week), cetuximab (1 mg BIW), or a combination of cetuximab and afatinib. **A.** Tumor volume and weight (inset) measurements. No treatment caused any significant reduction in animal weight. **B.** Area under curve analysis. There were five mice in each group. Treatment started 12 days after implantation. Groups were compared by ANOVA with Tukey's multiple comparisons test. Adjusted p-values are given. There were four animals per group.

Disease Models & Mechanisms • Supplementary information

Table S1. Demographic, clinical and other characteristics of patient samples and models

Click here to download Table S1

Table S2. Cytogenic characterization of DSRCT cell lines

Click here to download Table S2

Table S3. Materials used in this study

Click here to download Table S3

Disease Models & Mechanisms • Supplementary information
